# Strength of Products Made of Ultrafine-Grained Titanium for Bone Osteosynthesis

**DOI:** 10.3390/ma15238403

**Published:** 2022-11-25

**Authors:** Gennadiy V. Klevtsov, Ruslan Z. Valiev, Luiza R. Rezyapova, Natal’ya A. Klevtsova, Maksim N. Tyurkov, Mikhail L. Linderov, Maksim V. Fesenyuk, Olesya A. Frolova

**Affiliations:** 1 Department of Nanotechnology, Materials Science and Mechanics, Togliatti State University, 14 Belorusskaya St., 445020 Togliatti, Russia; 2 Institute of Physics of Advanced Materials, Ufa University of Science and Technology, 32 Z. Validi St., 450076 Ufa, Russia; 3 Laboratory of Multifunctional Materials, “Higher Engineering School of Aerospace Technologies” Center, Ufa University of Science and Technology, 32 Z. Validi St., 450076 Ufa, Russia; 4 Laboratory for Dynamics and Extreme Characteristics of Promising Nanostructured Materials, Saint Petersburg State University, 199034 St. Petersburg, Russia; 5 Research Institute of Progressive Technologies, Togliatti State University, 14 Belorusskaya St., 445020 Togliatti, Russia; 6 JSC “Production Association “Strela”, 26 Shevchenko St., 460005 Orenburg, Russia; 7 Department of the Mechanics of Materials, Structures and Machines, Orenburg State University, 13 pr. Pobedy, 460018 Orenburg, Russia

**Keywords:** coarse-grained (CG) and ultrafine-grained (UFG) Ti, strength, low-cycle fatigue, plates and screws for bone osteosynthesis

## Abstract

This paper evaluates the fatigue strength of ultrafine-grained (UFG) Grade 4 Ti in the low-cycle fatigue region, as well as the strength of medical implants (plates and screws) made of UFG Ti under various types of loading in comparison with the strength of products made of coarse-grained (CG) Ti. To produce a UFG state, titanium billets after annealing were processed by the ECAP-Conform technique. The fatigue of the prismatic specimens with a thickness of 10 mm from CG and UFG Ti was tested by the three-point bending method using an Instron 8802 facility. The modeling and evaluation of the stress-strain state in the ANSYS software package for finite-element analysis revealed, in particular, the localization of equivalent stresses in the area of hole edges and at fillets during the tension of the plates. The performed research has demonstrated that medical implants (plates and screws) from UFG Grade 4 Ti have a higher strength under different types of loading (tension, fatigue strength, torsion) in comparison with products from CG Ti. This opens up a possibility for the miniaturization of medical products from UFG Ti while preserving their main performance properties at an acceptable level.

## 1. Introduction

The current tendency for the miniaturization of medical products (plates and screws) for bone osteosynthesis in maxillofacial surgery, traumatology and several other areas of medicine presumes the use of materials that must have, in addition to high biocompatibility, a large set of mechanical properties [1,2,3,4]. In the process of operation, medical products for bone osteosynthesis may experience various loads in terms of both their value and loading type—static or cyclic. From this perspective, of great interest is the new class of bulk nanostructured metallic materials with an ultrafine-grained (UFG) structure produced by severe plastic deformation (SPD). There are a variety of SPD techniques, both well-established ones, such as high-pressure torsion, equal-channel angular pressing (ECAP) and ECAP-Conform [5,6], and new emerging ones (see, e.g., [7,8]). Numerous studies conducted in the past few years provide convincing evidence that the formation of a UFG structure enables increasing considerably the hardness and strength properties of materials during tension and torsion, and increasing the fatigue limit of materials [5,6,7,8,9,10,11,12,13,14]. The use of UFG materials that are stronger will enable miniaturizing medical implants without a decline in their mechanical properties under different types of loading. Of special interest is the evaluation of the fatigue strength of UFG materials for medical applications since the vast majority of medical products operate in the low-cycle fatigue region where, according to [15,16,17,18,19,20,21,22,23,24,25,26,27], SPD processing results in a decline or an insignificant increase in the fatigue strength of most structural materials. This in full measure refers to UFG Ti which, owing to its high biocompatibility and strength, is attractive for researchers in terms of manufacturing medical products for maxillofacial surgery, dentistry, and other areas of medicine [12,26,28]. Therefore, the issue of the effect of a UFG structure on the fatigue fracture resistance of Ti in the low-cycle fatigue region remains relevant.

The use of medical products from a new class of high-strength materials presumes the testing of finished products for strength under different types of loading. Such tests are performed in compliance with the general requirements stated in the Russian standard GOST R 15.013-2016 “System of products development and launching into manufacture. Medical devices”.

The aim of the present work is to evaluate the fatigue strength of UFG Ti for medical applications in the low-cycle fatigue region, and to evaluate comprehensively the strength of medical products from UFG Ti under different types of loading (plates under tension and cyclic loads; screws under torsion) in comparison with the strength of products from initial coarse-grained (CG) Ti.

## 2. Materials and Methods

As the material for the study in the CG and UFG states, Grade 4 Ti (0.003% N; 0.008% C; 0.0006% H; 0.32% O; 0.38% Fe, the rest is Ti), widely applied in medicine, was used. Ti in the CG state was studied after the homogenizing annealing of the hot-rolled billets at a temperature of 680 °C, 1 h. To produce a UFG state, the billets after homogenizing annealing were subjected to ECAP-Conform (ECAP-C) at a temperature of 200 °C (route Bc, *n* = 6, φ = 120°) and subsequent drawing (ECAP-C + D) at a temperature of 200 °C.

The structure formation in Grade 4 Ti during ECAP processing was examined in detail in [12]. The mean grain size and mechanical properties of CG and UFG Ti during tension and torsion are presented in Table 1.

The hardness of CG and UFG Ti was tested using a TH 300 hardness tester. The static tension of the cylindrical specimens with a diameter of 3 mm was conducted at a temperature of 20 °C using an H50KT universal testing machine. The fatigue tests of the prismatic specimens with a thickness of 10 mm were performed by the three-point bending method using an Instron 8802 facility. The fatigue tests of the specimens were conducted at a temperature of 20 °C with a loading frequency of ν = 10 Hz, a load ratio of R = 0.1 and different values of load ΔP, N. The fatigue fracture surface microrelief of the specimens was studied using a JEOL JCM-6000 scanning electron microscope (SEM), and the microrelief of the fracture surfaces produced during torsion was studied using a Phenom ProX G6 SEM.

**Figure 1:**
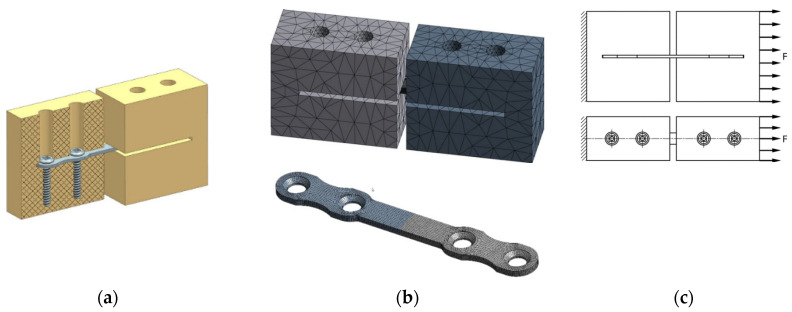
Solid (**a**) and mesh (**b**) models of a plate with bone fragments, and the respective loading principle (**c**).

**Figure 2:**
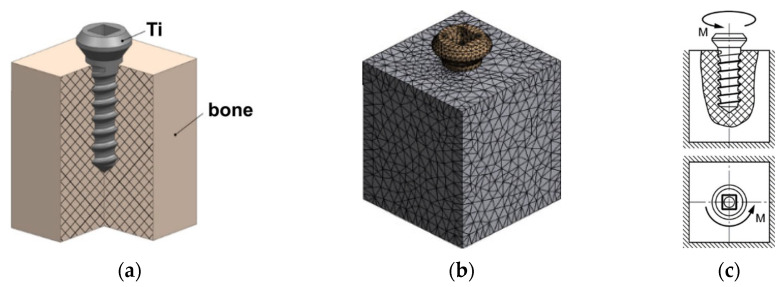
Solid (**a**) and mesh (**b**) models of a screw with a bone fragment, and the respective loading principle (**c**).

Prior to the strength test of the plates and screws, the modeling and evaluation of the stress–strain state of the elements of the investigated medical products was performed. On the basis of the developed models, the respective finite-element (FE) models were formed in the ANSYS Workbench software environment. The formation of FE models included generating a finite-element mesh, forming models of materials, specifying boundary conditions, modeling and specifying a load. To design the mesh models of plates, an unstructured mesh was generated using quadratic elements of a tetrahedral shape (Figure 1 and Figure 2). The mesh model of plates included 355,897 elements and 558,631 nodes, and the mesh model of screws included 77,493 elements and 128,301 nodes. The area of contact of screws with a plate is the mating conical surface of a screw head with a circular hole edge in a plate, the screw and the hole being coaxial.

The static calculation performed on the basis of the prepared FE models aimed to evaluate the stress–strain state in different zones of the plates. The force value in the calculation was varied in a range of 120 to 1200 N.

When specifying the models of materials, the finite elements characterizing a bone fragment were in all cases associated with an elasto-plastic model of the cortical bone, the parameters of which were given when describing previous calculations. The elements characterizing screws were in turn associated with two models of the material.

**Figure 3:**
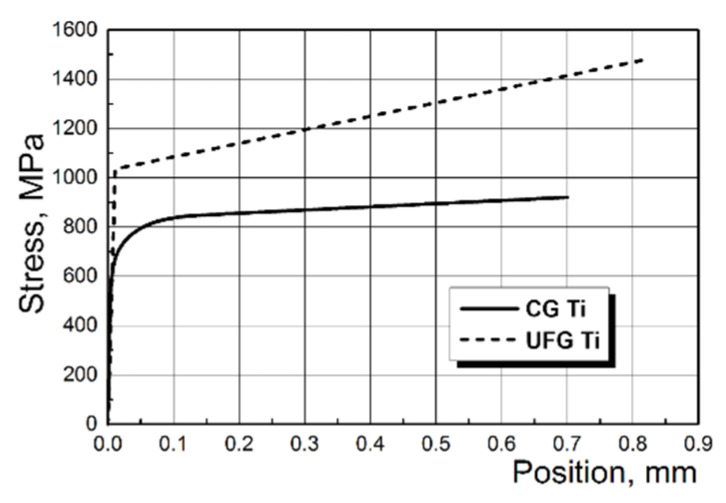
Titanium deformation curves used to determine the elasto-plastic models of titanium.

In the first variant, it was a linearly elastic model of titanium determined by the elastic modulus E and Poisson’s ratio μ (E = 105 GPa, μ = 0.34 for CG Ti, and E = 100 GPa, μ = 0.33 for UFG Ti). In the second case, it was an elasto-plastic model of titanium with bilinear isotropic hardening for UFG Ti and multi-linear isotropic hardening for CG Ti. When determining the respective models, the diagrams of material deformation (Figure 3) were specified on the basis of experimental data.

The interaction between the components of a model was considered through the creation of respective models of junctions (contact pairs). For the screw–bone interfaces, a bonded contact type was specified that implied a rigid connection between the finite elements in the interface surfaces of the mating bodies. For the screw–plate, plate–bone, and bone–finger interfaces, a standard (sliding) contact with friction was specified. The friction coefficients are presented in Table 2.

During the tensile and fatigue tests of the plates from CG and UFG Ti, we used a die-set and loading principles maximally close to the principles used in the modeling (Figure 1), where the plates were fastened with medical screws from CG and UFG Ti, respectively, in a die-set simulating a bone. The section of the plates from CG and UFG Ti in the central part was 0.6 × 2.6 mm. The tests were performed using an Instron ElectroPuls E1000 facility. In the tensile tests, the plates were loaded to failure. For the fatigue tests of the plates from CG and UFG Ti, identical loading parameters were selected: the cycle form was sinusoidal, the load ratio was R = 0.1, the loading frequency was ν = 20 Hz, and the load was ΔP = 180 N. The total number of loading cycles to failure was determined for the plates. The mechanical tests for the torsion of the screws from CG and UFG Ti were performed on a KTC-405-20-0.5 testing machine in compliance with the standards GOST R 50581-93 (ISO 6475-89) and GOST R 50582-93 (ISO 2835-91) [29,30]. Torque was applied in such a manner that the screw head rotated with a uniform angular rate of 1 rev. per minute with a gradual increase in torque until the screw fracture. The torque (N·m) and the rotation angle of the screw head (deg.) were recorded automatically with the building of a diagram and finding the maximum torque and rotation angle during the fracture of the screw.

## 3. Results

### 3.1. Fracture Kinetics and Mechanism of UFG Ti in the Low-Cycle Fatigue Region

**Figure 4:**
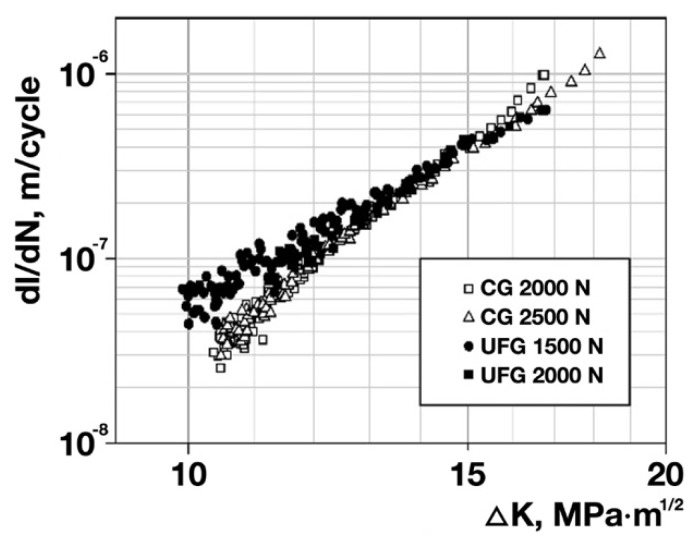
Straight-line portion of the kinetic diagrams of fatigue fracture for CG Ti (bright dots) and UFG Ti (dark dots) at different values of the load ΔP, N.

The fatigue tests of the specimens enabled building the kinetic diagrams of fatigue fracture for CG and UFG Ti (Figure 4). Analysis of the obtained diagrams shows that at the same value of the coefficient ΔK of stress intensity in a crack mouth, the fatigue crack propagation rates in CG and UFG Ti are approximately equal to each other (at least in the central part of the straight-line portion of the kinetic diagrams) (Figure 4). The coefficient n in the Paris equation describing the straight-line portion of the kinetic diagrams is lower for UFG Ti than for CG Ti (5.2 vs. 6.8) (Table 3).

**Figure 5:**
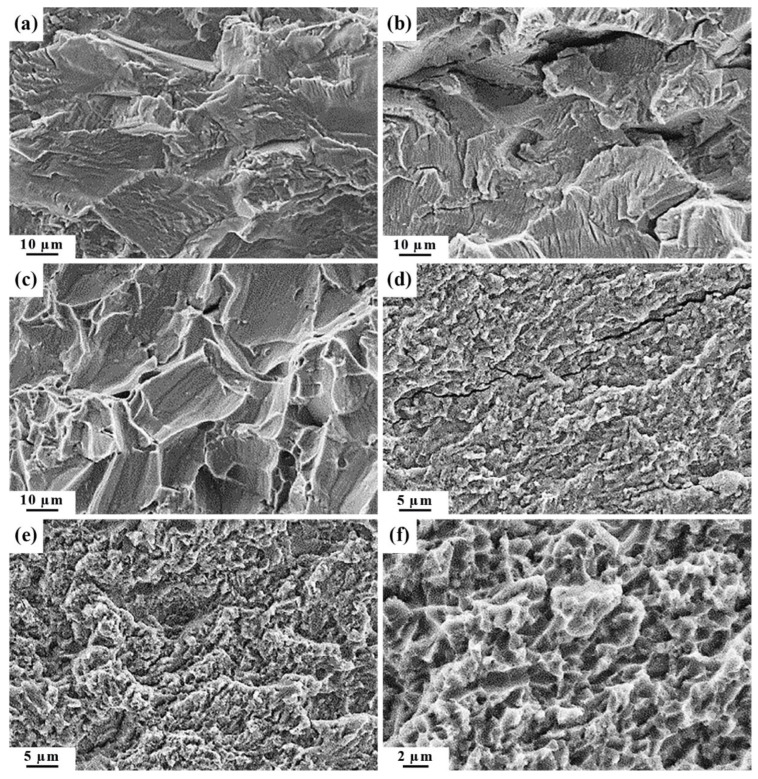
Fatigue fracture surface microrelief of the specimens from CG (**a**–**c**) and UFG (**d**–**f**) Ti: (**a**,**d**) in the vicinity of the crack initiation nucleus; (**b**,**e**) in the vicinity of the final failure zone; (**c**,**f**) in the final failure zone; (**a**–**c**) ×1000, (**d**,**e**) ×2000; (**f**) ×4000.

All the obtained fatigue fracture surfaces of the specimens, irrespective of the state of Ti, are even and smooth. On the fracture surfaces, two zones can be distinguished: the zone of a crack’s fatigue propagation, l_f_, and the zone of final failure [31,32]. Microfractographic analysis shows that in the vicinity of the fatigue crack initiation nucleus in CG Ti, one can observe flat fragments (Figure 5a) consisting of transcrystalline cleavage-like facets of approximately the same size as the grain size of CG Ti. In the central part of the l_f_ zone and closer to the final failure, well visible are fatigue striations and secondary cracks (Figure 5b). In the zone of final failure, fracture occurred via slip planes; at grain boundaries, there are areas with a microrelief close to a dimple one (Figure 5c). The fatigue fracture of UFG Ti at all the crack propagation stages is characterized by the formation of a fine microrelief. Visible are ductile fatigue striations and secondary cracks (Figure 5d,e). The final failure occurred with the formation of a dimple microrelief with small areas of quasi-cleavage (Figure 5e).

### 3.2. Tensile and Fatigue Strength Tests of the Plates

**Figure 6:**
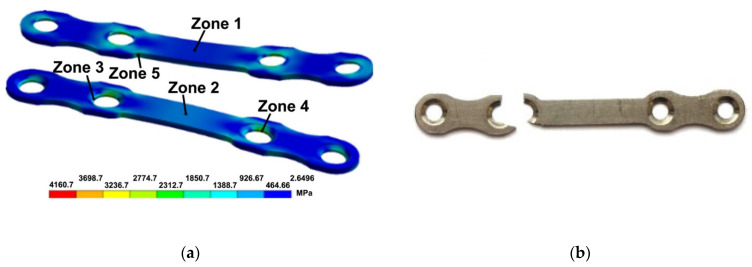
Localized equivalent stresses emerging in different zones of the plates from Ti during tension (**a**) and the characteristic view of the UFG Ti plates after tensile tests (**b**). Zones 1 and 2 are located in the central region of the top and bottom parts of the plates; zones 3 and 4 are located at hole edges; zone 5 is located at the fillets of the plates.

The results of the modeling and evaluation of the stress–strain state in the plates during tension show, in particular, the localization of equivalent stresses observed in the areas of hole edges and at fillets (zones 4, 5) (Figure 6a) in the plates from both CG and UFG Ti. Such local stresses may be the cause of plate fracture both during static tension and in the case of the cyclic loading of plates from the maximum tensile cycles.

**Figure 7:**
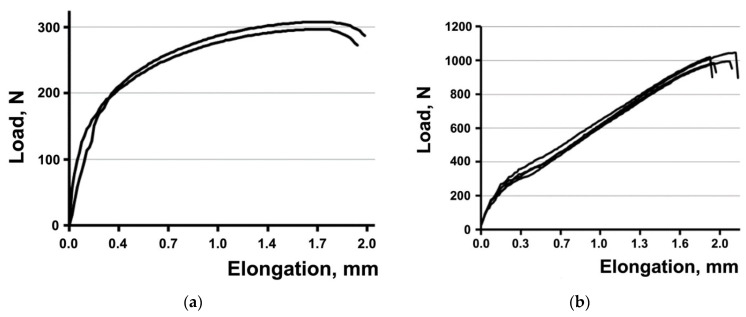
Tensile curves for the plates from CG (**a**) and UFG (**b**) Ti.

The tensile mechanical tests of the plates show (Figure 7a,b) that the UFG Ti plates withstood a stress 3.5 higher than the CG Ti plates (Table 4). At the same time, the elongation to failure of the plates from CG and UFG Ti is practically the same (Table 4). As noted above, the modeling results show the localization of equivalent stresses emerging under tensile stresses at hole edges and fillets in the plates. The performed tensile tests of the plates confirm the presence of weak sites in the plates in the area of holes where the fracture of all the plates occurred (Figure 6b). Therefore, the above-mentioned elongation of the plates has a purely conventional value.

The results of the fatigue tests of the plates show that the UFG Ti plates withstood a number of loading cycles to failure 2.8 larger than the CG Ti plates (Table 5). As in tensile tests, all the plates, irrespective of the state of Ti, fractured during fatigue loading in the area of holes. The latter is apparently conditioned by the maximum tensile cycles of loading.

### 3.3. Torsion Tests of the Screws

**Figure 8:**
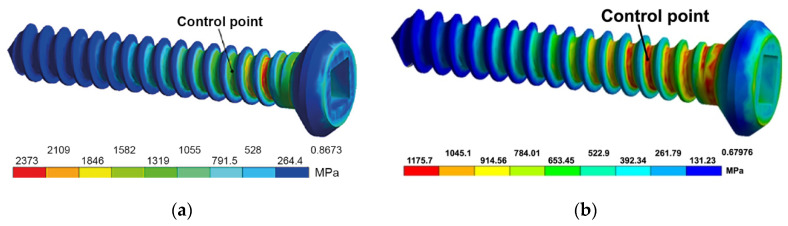
Equivalent stress in a UFG Ti screw for its elastic (**a**) and elasto-plastic (**b**) models.

The modeling of the stress–strain state in the screws from CG and UFG Ti during torsion revealed the presence of considerable equivalent stresses in the area between the head and the threaded portion (Figure 8) that may lead to the shear of screws. Calculation shows that the dependence of equivalent stresses on torque in the range of 0 to 0.24 N·m is practically linear. When torque is equal to 0.29 N·m, the equivalent stresses reach values close to the ultimate strength of CG and UFG Ti.

**Figure 9:**
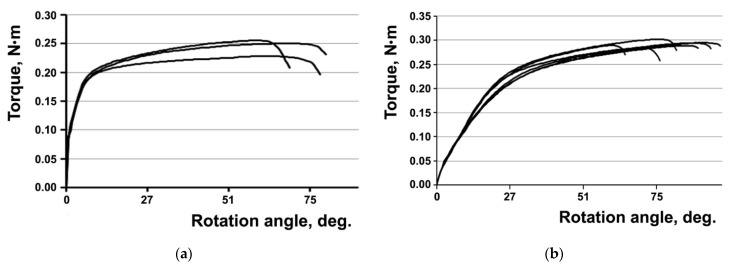
“Torque—rotation angle” diagrams based on the torsion tests of the screws from CG (**a**) and UFG (**b**) Ti.

The results of the torsional mechanical tests of the screws show (Figure 9a,b) that the torque leading to the fracture of UFG Ti screws is 1.2 times higher than the torque of the CG Ti screws. That being said, the rotation angles of the screws from CG and UFG Ti are approximately equal to each other (Table 6).

**Figure 10:**
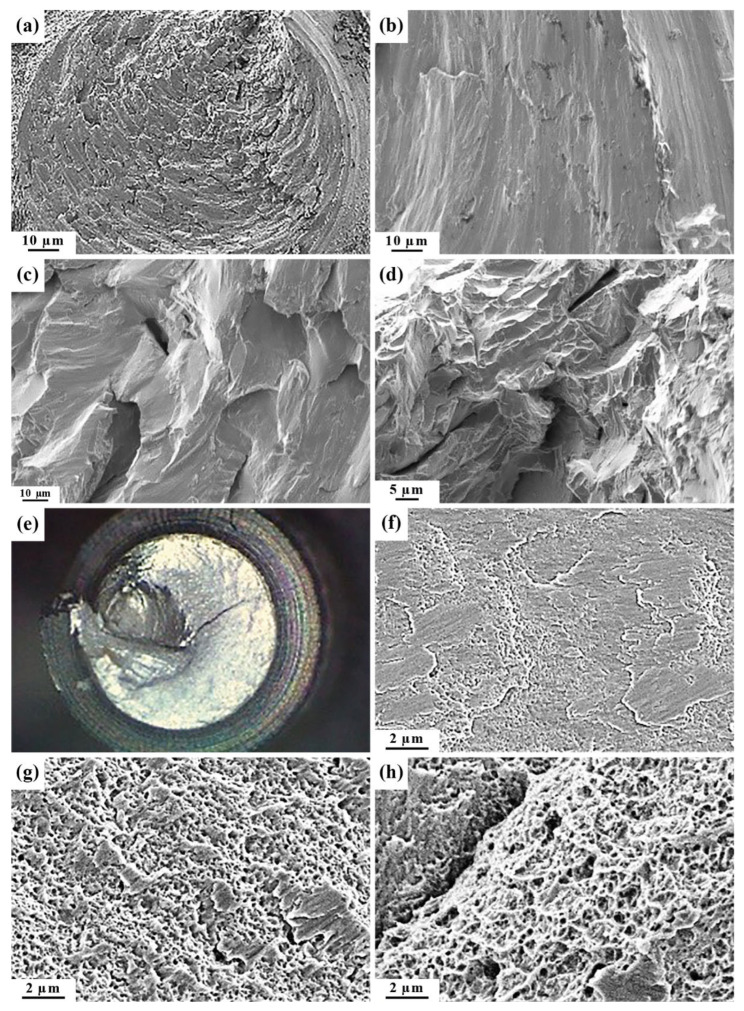
Characteristic view (**a,e**) and microrelief of the fracture surfaces for the screws from CG (**a**–**d**) and UFG (**e**–**h**) Ti. The microrelief was obtained in the peripheral (**b**,**f**), transitional (**c**,**g**) and central (**d**,**h**) parts of the fracture surfaces; (**b**) ×1000; (**c**) ×850; (**d**) ×750; (**f**–**h**) ×6000.

Irrespective of the state of Ti, three regions can be distinguished in all the fracture surfaces of the screws: the roughest central part slightly displaced from the fracture center, the transitional (middle) part and the relatively smooth peripheral part (Figure 10a,e). The microrelief of the peripheral part of the fracture surfaces for the screws from both CG and UFG Ti is a weak-relief one and has obvious signs of rubbing out (Figure 10b,f) that resulted from the mutual friction between the mating fracture surfaces during screw torsion. In the middle part of the fractures of the CG Ti screws (Figure 10c), the microrelief consists of cleavage-like flat fragments that are partially rubbed out; secondary cracks can be seen. The microrelief of this part of the fracture surfaces for the UFG Ti screws consists of small shear dimples (Figure 10g). In the central part of the fracture surfaces for the CG Ti screws (Figure 10d), fracture occurred via the quasi-cleavage mechanism, and for the UFG Ti screws, fracture occurred with the formation of small equiaxed rupture dimples (Figure 10h).

Thus, analysis of the microrelief of the fracture surfaces of the screws shows that during the torsion of the screws from CG and UFG Ti, fracture starts in the peripheral part of the screws via shear. Fracture in the central part of the screws occurs via rupture.

## 4. Discussion

As noted above, medical products (plates and screws) for bone osteosynthesis used in maxillofacial surgery, traumatology, and other areas of medicine experience significant and various loads, depending on their application, in the process of operation.

Analysis of the operational damages of medical products shows [4] that the fracture of plates for bone osteosynthesis is mainly related to tensile static loads and cyclic loads in the low-cycle fatigue region. The fracture of screws for the fixation of plates and bone fragments in most cases takes place via the shear of a screw between the threaded portion and the head. This happens most often during the unscrewing of screws fused with bone conducted after the recovery of a patient or due to other reasons [4,33].

The higher strength properties of UFG Grade 4 Ti during tension, as compared with CG Ti (Table 1), undoubtedly have a beneficial effect on the static tension resistance of the plates for bone osteosynthesis. This is convincingly demonstrated by the results of the tensile mechanical tests of the plates showing that the plates from UFG Ti withstood a stress 3.5 times higher than that of the plates from CG Ti (Figure 7 and Table 4). However, the presence of weak sites in the plates of a classic shape in the area of hole edges and at fillets, discovered during modeling (Figure 6a) and confirmed experimentally (Figure 6b) indicates the need for a serious improvement of the plate designs.

The high strength properties during tension of UFG materials do not always have a beneficial effect on the fatigue strength of materials in the low-cycle fatigue region [14,24]. To characterize the fatigue fracture resistance of a material in the low-cycle fatigue region, the analysis of the kinetic diagrams of fatigue fracture is currently used [34]. The performed fatigue tests of the specimens from CG and UFG Grade 4 Ti show that at the same value of the stress intensity coefficient Δ*K*, the fatigue crack propagation rates in CG and UFG Ti in the middle part of the kinetic diagram are approximately equal to each other (Figure 4). However, the coefficient n in the Paris equation describing the straight-line portion of the kinetic diagrams is lower for UFG Ti than for CG Ti (5.2 vs. 6.8) (Table 3). Consequently, UFG Ti is less sensitive to cyclic overloads [31,32] emerging during the operation of medical products. The slower increment in the fatigue crack propagation rate with increasing stress intensity coefficient Δ*K* and the lower sensitivity to cyclic overloads of UFG Ti are apparently associated with the more ductile character of the fatigue fracture of UFG Ti in comparison to CG Ti (Figure 4). This fact has had a beneficial effect on the fatigue strength of the plates from UFG Ti.

The fatigue test results for the plates demonstrate that at the same geometrical sizes of the plates and test conditions, the total number of loading cycles to failure for the plates from UFG Ti is 2.8 times larger than that for the plates from CG Ti (Table 5). However, like in the tensile tests of the plates, during fatigue loading all the plates, irrespective of the state of Ti, fractured near holes. This fact should also be considered during the future development of the plate designs.

In [12,13], it is shown through the example of Ti and austenitic steel that materials with a UFG structure resist torsional fracture better than initial CG materials. However, bone screws are complex-shaped items with a pronounced stress raiser (thread). The torsional mechanical tests of the screws demonstrate (Figure 9) that the torque leading to the fracture of the screws from UFG Ti is slightly (1.2 times) higher than the torque of the screws from CG Ti. That being said, the rotation angles of the screws from CG and UFG Ti are approximately equal to each other (Table 6).

Thus, the performed research demonstrates that the medical products (plates and screws) made of UFG Grade 4 Ti have a higher strength under different types of loading (tension, fatigue strength, torsion) in comparison with the products made of CG Ti. This opens up a possibility for the miniaturization of medical products from UFG Ti while preserving their main performance properties at an acceptable level.

## 5. Conclusions

1. Analysis of the kinetic diagrams of fatigue fracture for CG and UFG Ti shows that at the same stress intensity coefficient ΔK, the fatigue crack propagation rates of CG and UFG Ti in the middle part of the kinetic diagram are approximately equal to each other. The coefficient n in the Paris equation describing the straight-line portion of the kinetic diagrams is lower for UFG Ti than for CG Ti (5.2 vs. 6.8). Consequently, UFG Ti is less sensitive to cyclic overloads emerging during the operation of medical products.
2. The tests of the plates show that the UFG Ti plates withstood a stress 3.5 higher than the CG Ti plates and a number of loading cycles to failure 2.8 larger than the CG Ti plates.
3. The results of the modeling and evaluation of the stress–strain state in the plates during tension show, in particular, the localization of equivalent stresses observed in the areas of hole edges and at fillets in the plates from both CG and UFG Ti. The performed tensile tests of the plates confirm the presence of weak sites in the plates in the area of holes where fracture occurred in all the plates subjected to both tensile and fatigue strength tests.
4. The torsion mechanical tests of the screws show that the torque leading to the fracture of the UFG Ti screws is slightly (1.2 times) higher than the torque of the CG Ti screws. That being said, the rotation angles of the screws from CG and UFG Ti are approximately equal to each other.

The performed research demonstrates that the medical implants (plates and screws) made of UFG Grade 4 Ti have higher strength properties under different types of loading in comparison with the products made of CG Ti. This opens up a possibility for the miniaturization of medical products from UFG Ti while preserving their main performance properties at an acceptable level.

## Figures and Tables

**Table 1:** Mean grain size and mechanical properties of Grade 4 Ti during tension and torsion [12].

State	d_mean,_ μm	HB	σ_B_, MPa, during Tension	σ_0.2_, MPa, during Tension	δ, %, during Tension	τ_k,_ MPa, during Torsion	τ_0.3_, MPa, during Torsion	g, %, during Torsion
Coarse-grained (CG), annealing	25	255	750 ± 10	650 ± 30	20 ± 0.5	920 ± 37	641 ± 46	154 ± 3.5
Ultrafine-grained (UFG), ECAP-C + D	0.2	293	1250 ± 10	1100 ± 30	11 ± 0.5	1014 ± 24	831 ± 18	87 ± 2.0

**Table 2:** Friction coefficients for junctions.

Titanium–Titanium Junction	Titanium–Bone Junction	Steel–Bone Junction
k = 0.36	k = 0.3	k = 0.36

**Table 3:** Paris equations describing the straight-line portion of the kinetic diagrams of fatigue fracture for CG and UFG Ti.

State	Equation
CG Ti	$\frac{dl}{dN}=4.2\cdot 10^{-15}{\left(\Delta K\right)}^{6.8}$
UFG Ti	$\frac{dl}{dN}=43\cdot 10^{-13}{\left(\Delta K\right)}^{5.2}$

**Table 4:** Plate fracture parameters.

Material	Load, N	Elongation, mm
CG Ti	302 ± 5.0	1.95 ± 0.05
UFG Ti	1011 ± 12	2.04 ± 0.13

**Table 5:** Average plate life.

Material	Number of Cycles to Failure
CG Ti	(4.6 ± 2.0) × 10^3^
UFG Ti	(1.3 ± 0.3) × 10^4^

**Table 6:** Average values of the parameters of screw fracture during torsion.

Material	Torque, N·m	Rotation Angle, deg.
CG Ti	0.24 ± 0.01	69 ± 8
UFG Ti	0.29 ± 0.01	80 ± 15

## Data Availability

Not applicable.
